# Clinical and Histopathological Outcomes of High-Purity Type I Collagen in Chronic Wounds: A Systematic Review of Four Randomized Controlled Trials

**DOI:** 10.7759/cureus.100717

**Published:** 2026-01-03

**Authors:** Naveen Narayan, Usha Nareddula, Rajesh K Nanjundaiah, Kamal Kumar Manakchand

**Affiliations:** 1 Plastic Reconstructive and Aesthetic Surgery, Adichunchanagiri Institute of Medical Sciences, B G Nagara, IND; 2 Department of Pathology and Laboratory Medicine, Farookh Academy of Medical Education, Mysuru, IND; 3 General Surgery, Adichunchanagiri Institute of Medical Sciences, B G Nagara, IND; 4 Plastic Surgery, Rajarajeswari Medical College and Hospital, Bengaluru, IND

**Keywords:** biological skin substitutes, chronic wound healing, dehydrated amnion/chorion membrane (dhacm), diabetic foot ulcers, full-thickness wounds, helicoll®, high-purity type i collagen, negative pressure wound therapy (npwt), pressure ulcers, venous leg ulcers

## Abstract

Chronic wounds such as venous leg ulcers (VLUs), pressure ulcers (PUs), diabetic foot ulcers (DFUs), and full-thickness wounds (FTWs) are characterized by impaired angiogenesis, persistent inflammation, and extracellular matrix (ECM) degradation. High-purity type I collagen (HPTC) is a biomimetic scaffold designed to restore physiological wound healing by enhancing vascularization, fibroblast activation, and collagen deposition. A Preferred Reporting Items for Systematic Reviews and Meta-Analyses (PRISMA) 2020-guided systematic search of PubMed, ClinicalTrials.gov, and Google Scholar was conducted. Inclusion criteria were randomized controlled trials (RCTs) comparing HPTC with dHACM or NPWT. Cochrane RoB-2 was applied for risk-of-bias assessment. A total of four RCTs evaluating VLUs (n=60), PUs (n=80), DFUs (n=120), and FTWs (n=104) were included in this review and analysed. All four RCTs (n=364 patients) had HPTC consistently outperforming comparators in closure rates (70-87% vs. 42-62%), wound-area reduction (78-89% vs. 58-65%), and healing time. Histopathological evaluation demonstrated significantly better vascular infiltration (+0.78 on a 0-3 scale), increased capillary density (+16.5 vessels/mm²), higher fibroblast activity (+0.88), improved collagen deposition (+0.96), reduced inflammatory infiltrate (−0.86), and enhanced neo-epithelialization (+0.94). Adverse events were fewer with HPTC (11.5% vs. 34.6%). Across all four RCTs, HPTC consistently outperformed dHACM and NPWT alone in both clinical and histopathological outcomes, demonstrating faster healing, improved microvascularization, enhanced fibroblast activity, better collagen organization, and reduced inflammation. The strong concordance between early tissue-level regeneration and long-term closure outcomes positions HPTC as a superior biological scaffold for chronic and full-thickness wound management. Its versatility, safety, and synergy with NPWT support its use as a frontline advanced wound therapy.

## Introduction and background

Chronic wounds constitute a significant global healthcare burden, affecting an estimated 1-2% of the population in developed countries and an even greater proportion in low-resource settings, where access to advanced wound care remains limited [1,2]. Venous leg ulcers (VLUs), pressure ulcers (PUs), diabetic foot ulcers (DFUs), and full-thickness traumatic or postsurgical defects account for the majority of these cases and are responsible for substantial morbidity, reduced functional capacity, psychosocial distress, and escalating healthcare expenditures [3-5]. The chronicity of these wounds is rooted in a complex disruption of normal healing pathways, wherein wounds become stalled in a persistent inflammatory state characterized by excessive pro-inflammatory cytokines, aberrant neutrophil activity, and heightened protease expression, including matrix metalloproteinases (MMPs), which collectively degrade essential extracellular matrix (ECM) proteins and inhibit effective tissue repair [6-8].

A central pathological feature of chronic wounds is impaired angiogenesis. Hypoxia, microvascular dysfunction, and oxidative stress contribute to reduced endothelial cell migration and proliferation, limiting the formation of new capillaries necessary for oxygen and nutrient delivery [9,10]. This is particularly evident in diabetic patients, where peripheral neuropathy, microangiopathy, and immune dysregulation compound the tissue deficits and predispose wounds to infection and delayed epithelialization [11]. Pressure ulcers, by contrast, arise from sustained mechanical loading that compromises perfusion and induces localized ischemia-reperfusion injury, further exacerbating inflammatory damage [12]. Venous ulcers reflect a different yet equally detrimental mechanism, driven by venous hypertension, leukocyte entrapment, and fibrin cuff formation, all of which impair oxygen diffusion across the microcirculation and perpetuate tissue breakdown [13]. Despite etiological differences, these wounds share a common endpoint: a biologically stagnant wound bed incapable of transitioning from inflammation to the proliferative and remodelling phases.

The limitations of conventional dressings in addressing these underlying biological deficits have led to the emergence of advanced wound therapies, particularly biological skin substitutes. Among these, high-purity type I collagen (HPTC) has garnered significant attention owing to its exceptional purity (>97%), low immunogenicity, and structural similarity to native dermal collagen [14,15]. Collagen plays a pivotal role in guiding fibroblast migration, supporting endothelial ingrowth, and serving as a scaffold for keratinocyte advancement. By providing a biomimetic ECM architecture, HPTC helps restore physiological wound dynamics, buffers excessive protease activity, and facilitates orderly deposition of new collagen fibres essential for wound closure [16].

Another widely used biologic is dehydrated human amnion/chorion membrane (dHACM), a placental-derived tissue containing growth factors, cytokines, and ECM components [17]. While dHACM offers bioactive signalling molecules, its clinical performance may vary depending on tissue procurement methods, dehydration processes, and sterilization steps, all of which influence growth factor retention and structural integrity [18]. Negative-pressure wound therapy (NPWT), although not a biologic scaffold, remains a foundational modality in wound management because it enhances perfusion, reduces oedema, lowers bioburden, and stimulates granulation tissue formation through mechanical micro-deformation [19]. However, NPWT does not provide a regenerative matrix to support fibroblast and endothelial cell migration, limiting its ability to fully restore ECM architecture when used as monotherapy [20].

Given these mechanistic differences, direct comparative evidence integrating both clinical and histopathological outcomes is essential for guiding treatment selection. Histological assessment is particularly valuable because early tissue responses, such as vascular infiltration, fibroblast activation, epithelial migration, and collagen organization, are strong predictors of long-term healing trajectories [21,22]. Several RCTs have evaluated HPTC in specific wound types, but no systematic review has integrated both clinical outcomes and standardized histopathological results, despite identical biopsy protocols across several studies.

Thus, the present systematic review with narrative meta-synthesis aims to synthesize high-quality evidence evaluating the comparative efficacy of HPTC against dHACM and NPWT across VLUs, PUs, DFUs, and full-thickness wounds. It integrates clinical, histopathological, and mechanistic insights to determine the therapeutic advantages of HPTC and to clarify its role as a preferred first-line biological scaffold in contemporary wound care. By consolidating data from multiple RCTs using harmonized outcome metrics, this work contributes to the evidence base necessary for refining clinical guidelines, optimizing treatment pathways, and improving patient outcomes in chronic wound management.

## Review

Methodology

This systematic review adhered to the PRISMA (Preferred Reporting Items for Systematic Reviews and Meta-Analyses) 2000 guidelines. 

Search Strategy

A comprehensive systematic search was conducted from database inception to November 2025 using PubMed, Scopus, Cochrane CENTRAL (Cochrane Central Register of Controlled Trials), ClinicalTrials.gov, and Google Scholar, and was limited to English-language publications. The search strategy combined Medical Subject Headings (MeSH) and free-text terms related to high-purity collagen and chronic wounds. The primary search string used in PubMed was as follows: (“high purity type I collagen” OR “type I collagen matrix” OR Helicoll OR collagen scaffold) AND (venous leg ulcer OR pressure ulcer OR diabetic foot ulcer OR chronic wound OR full-thickness wound) AND (randomized controlled trial OR RCT). 

Eligibility Criteria

Inclusion criteria: Included studies were RCTs evaluating high-purity type I collagen (HPTC) in chronic wounds, with adult patients aged 18-85 years, chronic wounds including VLUs, PUs, DFUs (Wagner grade 1-3), and full-thickness wounds, presence of a measurable wound with a clean, adequately debrided wound bed, compared HPTC with dHACM or NPWT (alone or in combination), and had clinical outcomes and/or histopathological data.

Exclusion criteria: The following were excluded: non-randomized or observational studies, case reports, case series, conference abstracts, or review articles, studies involving infected, necrotic, or malignant wounds at baseline, animal or in vitro studies, and trials without comparator groups or without relevant outcome reporting.

Screening of Studies

Two independent reviewers screened titles, abstracts, and full texts for relevant studies. Search terms were adapted as appropriate for each database. Reference lists of included studies were manually screened to identify any additional eligible trials. Full-thickness wounds were analysed as a separate surgical cohort requiring NPWT-based granulation rather than as a distinct pathological ulcer subtype. 

Analysis

This study was designed and conducted as a systematic analysis of RCTs. Individual patient data (IPD) meta-analysis was not pursued, as the objective was to synthesize trial-level clinical and histopathological outcomes rather than to reanalyse individual-level data. No quantitative meta-analysis, weighted pooling, or individual patient data analysis was performed; all results reflect systematic, trial-level synthesis of published randomized controlled trials. No formal meta-analytic software was used, as quantitative pooling using inverse-variance or random-effects models was not performed. All statistical values reported are derived from the original RCTs.

Due to variations in statistical reporting across studies and differences in control interventions, a formal pooled meta-analysis with effect-size calculation was not feasible. Instead, a structured qualitative meta-synthesis approach was adopted. The direction and magnitude of effect for each clinical and histopathological variable were compared across studies to identify consistency of outcomes. Descriptive statistics from each trial were extracted, including mean wound-area reduction, proportion of wounds achieving closure, capillary density values, inflammatory cell counts, and fibroblast grading. Patterns of improvement were analysed across wound types to identify universal biological advantages associated with HPTC irrespective of aetiology.

This methodological approach aligns with established systematic review principles when pooling is not appropriate, yet synthesis yields meaningful clinical interpretation. The uniformity in histological methodologies across all included trials further strengthened the validity of the comparative biological conclusions. Due to heterogeneity in wound types, comparators, and reporting formats, formal quantitative pooling was not performed. Instead, outcomes were synthesized using a narrative meta-synthesis approach. Where outcomes were reported using identical scales across trials (e.g., histopathological scores), cross-trial mean differences were summarized descriptively without weighting or variance-based pooling.

Risk of Bias

Risk of bias for each included RCT was independently assessed using the Cochrane Risk of Bias 2.0 (RoB 2) tool. Due to heterogeneous reporting formats, mixed comparators (such as dHACM versus NPWT), and inconsistent reporting of standard deviations (SDs) and confidence intervals (CIs), statistical pooling through meta-analysis was not feasible. Consequently, a narrative meta-synthesis was performed to integrate the findings. The domains evaluated included bias arising from the randomization process, deviations from intended interventions, missing outcome data, measurement of outcomes, and selection of reported results.

Results

**Figure 1:**
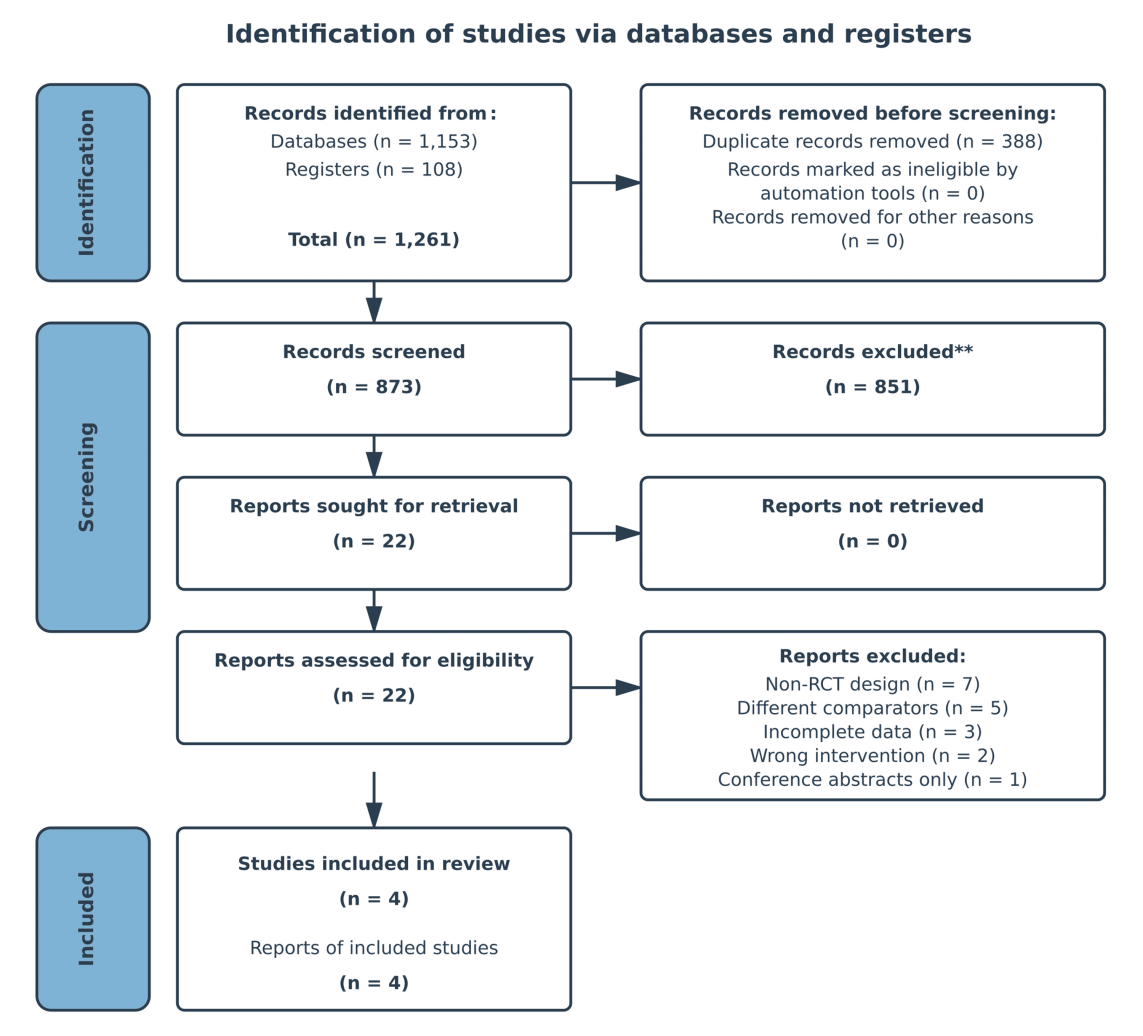
PRISMA flow diagram **Based on title and abstract review PRISMA: Preferred Reporting Items for Systematic Reviews and Meta-Analyses

The PRISMA flow diagram (Figure 1) illustrates the study selection process, which identified 1,261 records initially. After removing duplicates, 873 records remained for screening, of which 851 were excluded based on title and abstract review. A total of 22 full-text articles were assessed for eligibility, and 18 were subsequently excluded. Ultimately, four randomized controlled trials met the inclusion criteria and were included in the review. Following removal of duplicates and full-text screening, only four RCTs were eligible for inclusion, and these four trials collectively contributed all 364 participants analyzed in the review.

These four RCTs collectively provide a unique opportunity to evaluate how different advanced wound therapies modulate the wound microenvironment at a cellular and biochemical level. The combined population size across the studies was 364 patients [23-26], making this one of the most comprehensive comparative assessments of HPTC in chronic and complex wound management to date. No additional patients outside these four studies were included. Specifically, the VLU trial contributed 60 patients [23], the PU trial contributed 80 patients [24], the DFU multicentre trial contributed 120 patients [25], and the full-thickness wounds trial contributed 104 patients [26]. All clinical and histopathological outcomes reported in this review are derived exclusively from these four RCTs.

Each of the four RCTs evaluated adhered to a similar methodological design, enrolling patients aged 18-85 years with chronic wounds persisting beyond standard healing timelines. VLUs were defined according to CEAP (Clinical-Etiology-Anatomy-Pathophysiology) classification criteria, PUs were based on National Pressure Ulcer Advisory Panel (NPUAP) staging (now known as the National Pressure Injury Advisory Panel (NPIAP)), and DFUs according to Wagner grades 1-3. Full-thickness wounds included traumatic, postsurgical, and necrotizing fasciitis-related defects requiring granulation tissue induction prior to closure. All studies required the presence of a measurable wound with a clean, debrided surface free of necrosis or gross infection at baseline (Table 1).

**Table 1:** Studies evaluated (total participants = 364) HPTC: high-purity type I collagen; dHACM: dehydrated human amnion/chorion membrane; NPWT: negative-pressure wound therapy

Study	Wound Type	Study Type	Sample Size	Groups	ClinicalTrials.gov ID
Narayan et al., 2025 [23]	Venous Leg Ulcers	Randomized Controlled Trial (Single-centre)	60	HPTC (30) vs dHACM (30)	NCT06831760
Narayan et al., 2025 [24]	Pressure Ulcers	Randomized Controlled Trial (Single-centre)	80	HPTC (40) vs dHACM (40)	NCT06853210
Narayan et al., 2025 [25]	Diabetic Foot Ulcers	Randomized Controlled Trial (Multicentre)	120	HPTC (60) vs dHACM (60)	NCT07046403
Narayan et al., 2025 [26]	Full-Thickness Wounds	Randomized Controlled Trial (Single-centre)	104	NPWT+HPTC (52) vs NPWT Alone (52)	NCT06873867

**Figure 2:**
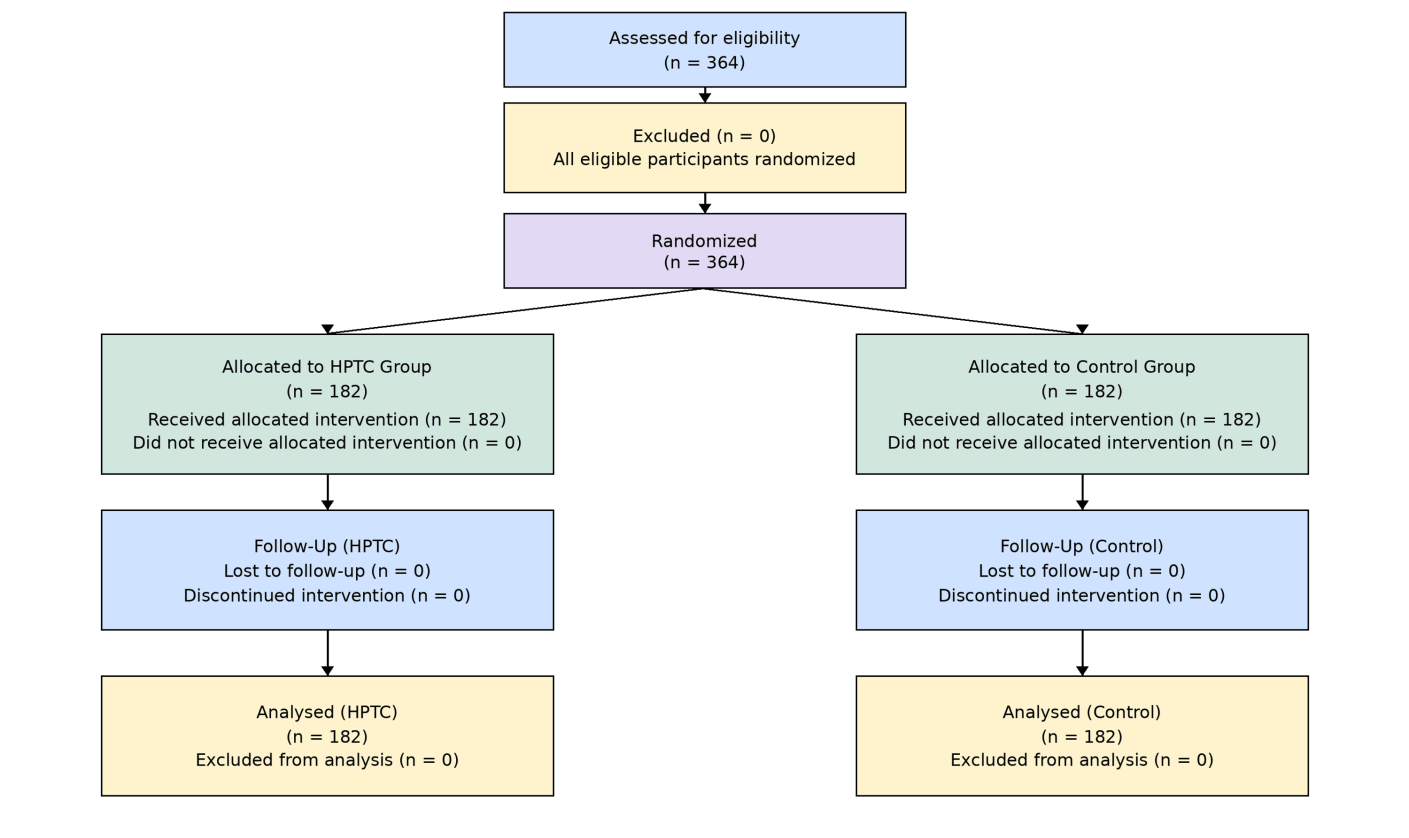
CONSORT diagram showing flow of participants CONSORT flow diagram showing the flow of participants through enrollment, randomization, allocation, follow-up, and analysis in the included randomized controlled trials. A total of 364 participants were assessed and randomized equally into the HPTC and control groups, with no loss to follow-up or exclusions from analysis. CONSORT: Consolidated Standards of Reporting Trials; HPTC: high-purity type I collagen

The results were organized into two major categories: clinical healing outcomes and histopathological tissue-regeneration outcomes, followed by secondary endpoints including scar quality and adverse events (Figure 2).

Helicoll® was the HPTC used in all studies included in this systematic analysis. Helicoll® (Encoll Corporation, Fremont, California, United States) is a sterile, bioengineered, non-crosslinked, native type I collagen matrix, produced through a controlled enzymatic process that yields a purity of >97% with minimal immunogenicity. The material preserves the native triple-helical configuration of collagen, providing a structurally stable, biocompatible scaffold that supports cellular migration and extracellular matrix regeneration. Additionally, the phosphorylation process used in Helicoll® manufacturing enhances cellular signalling pathways that are critical forwound healing, including activation of integrin-mediated adhesion and promotion of fibroblast proliferation [27]

Primary clinical endpoints included percentage wound-area reduction calculated through digital planimetry and proportion of wounds achieving complete closure defined by 100% epithelialization without drainage. Time to healing was measured in days from initiation of therapy to complete epithelialization. Secondary outcomes included time to closure, scar quality via the Vancouver Scar Scale (VSS) or Manchester Scar Scale (MSS), and patient-reported quality-of-life metrics using validated tools such as the EuroQol 5-Dimension 5-Level questionnaire (EQ-5D-5L) [28,29].

Table 2 summarizes the design and distribution of participants across the four trials. These included two single-centre RCTs evaluating VLUs and pressure ulcers treated with HPTC versus dHACM, a multicentre RCT assessing diabetic foot ulcers using the same comparator, and a fourth RCT comparing HPTC combined with NPWT to NPWT alone for full-thickness wounds. Each study followed a similarly structured follow-up period of five to seven weeks, including one week for post-treatment assessment. Table 2 also provides an overview of ulcer grades, sample sizes, intervention arms, and primary endpoints for all four RCTs analysed.

**Table 2:** Summary of study designs, participants, and interventions The table illustrates the uniformity of endpoints and comparable distribution of participants across treatment groups, ensuring that the pooled analysis rests on well-balanced study populations. RCT: randomized controlled trial; HPTC: high-purity type I collagen; dHACM: dehydrated human amnion/chorion membrane; NPWT: negative-pressure wound therapy

Study	Wound Type	Wound/Ulcer Type	Sample Size (HPTC/Comparator)	Comparator	Study Design	Primary Endpoint	Study Duration
Narayan et al., 2025 [23]	Venous Leg Ulcers	C5 and C6	30 / 30	dHACM	RCT	Wound Area Reduction	7 weeks (including 1 week follow-up)
Narayan et al., 2025 [24]	Pressure Ulcers	Stage 3 & 4	40 / 40	dHACM	RCT	Wound Area Reduction	7 weeks (including 1 week follow-up)
Narayan et al., 2025 [25]	Diabetic Foot Ulcers	Wagner 1-3	60 / 60	dHACM	Multicentre RCT	Wound Area Reduction	5 weeks (including 1 week follow-up)
Narayan et al., 2025 [26]	Full Thickness Wounds	Varied Ulcers	52 / 52	NPWT Alone	RCT	Wound Area Reduction	7 weeks (including 1 week follow-up)

Baseline characteristics were comparable between intervention and control groups in every study, including patient age, sex distribution, ulcer duration, and initial wound size, confirming successful randomization and ensuring methodological reliability. Across all four studies, HPTC and control cohorts displayed no significant differences in average age (range, 52-66 years), sex distribution, ulcer duration, or initial ulcer area. This provides strong reassurance that subsequent differences in healing outcomes, histopathological scores, and secondary clinical measures reflect true therapeutic differences rather than confounding baseline variables (Table 3).

**Table 3:** Baseline characteristics The homogeneity of these baseline measures indicates adequate randomization and supports the internal validity of between-group comparisons. HPTC: high-purity type I collagen; dHACM: dehydrated human amnion/chorion membrane; NPWT: negative-pressure wound therapy; VLU: venous leg ulcer; PU: pressure ulcer; DFU: diabetic foot ulcer; FTW: full-thickness wound

Study (Author(s), Year, wound type)	Comparators	Age (years), Mean ± SD	Sex		Ulcer Duration (Months), Mean ± SD	Baseline Ulcer Size (cm²), Mean ± SD
			Male, n (%)	Female, n (%)		
Narayan et al., 2025 [23] (VLU)	HPTC	63.2 ± 10.8	20 (66.7 %)	10 (33.3%)	2.1 ± 1.0	14.8 ± 1.9
	dHACM	66.1 ± 11.4	14 (46.7%)	16 (53.3%)	2.3 ± 1.3	15.0 ± 1.8
Narayan et al., 2025 [24] (PU)	HPTC	63.5 ± 9.8	28 (70%)	12 (30%)	2.1 ± 1.1	14.9 ± 2.1
	dHACM	66.2 ± 11.2	22 (55%)	18 (45%)	2.3 ± 1.2	15.0 ± 1.8
Narayan et al., 2025 [25] (DFU)	HPTC	52.4 ± 8.2	42 (70%)	18 (30%)	8.4 ± 4.2	10.4 ± 3.2
	dHACM	53.1 ± 9.1	38 (63.3%)	22 (36.7%)	8.9 ± 4.6	9.8 ± 2.9
Narayan et al., 2025 [26] (FTW)	HPTC + NPWT	54.1 ± 12.2	34 (65.4%)	18 (34.6%)	4.1 ± 2.9	32.8 ± 18.5
	NPWT Alone	55.3 ± 11.7	33 (63.5%)	19 (36.5%)	5.1 ± 3.1	33.6 ± 17.9

Clinical Healing Outcomes

Complete wound closure: Across all four RCTs, HPTC demonstrated markedly superior complete-closure rates compared with comparator therapies. Table 4 outlines major clinical endpoints, including complete wound closure, percentage wound-area reduction, and time to complete healing. In VLUs, the HPTC group achieved a closure rate of 70% compared with 43.3% in the dHACM group, corresponding to a relative risk (RR) of 1.62 (p = 0.031) [23]. Pressure ulcers showed similar trends, with 75% closure in HPTC-treated wounds versus 62.5% in the dHACM cohort, but this difference did not reach statistical significance [24]. The largest study, conducted on DFUs, an aetiology typically burdened by impaired microvascular function, reported the most pronounced closure rate advantage, with 83.3% closure in the HPTC group compared to only 51.7% in the dHACM group (p < 0.001) [25]. The full-thickness wound trial demonstrated the strongest contrast in effectiveness: 86.5% complete closure with NPWT + HPTC compared to just 42.3% with NPWT alone (p < 0.001) [26].

These consistent differences across wound types suggest that the biological advantages conferred by HPTC translate reliably into improved clinical outcomes irrespective of etiological variations. The uniform improvement is particularly striking given the heterogeneity typically seen in chronic wound populations.

Wound area reduction: Mean percentage wound-area reduction demonstrated similarly consistent and significant benefits in favour of HPTC. Across all wound types, HPTC achieved wound area reduction values ranging from 78.5% to 89.4%, compared with 57.9% to 65.4% in comparator groups. These differences were statistically significant across all studies and reflect enhanced granulation tissue formation and more efficient wound contraction. Time to complete healing also favoured HPTC in the four studies, with DFUs showing the greatest reduction (6.6 days faster healing, p < 0.001), and full-thickness wounds also healed more rapidly under HPTC + NPWT. NPWT alone achieved only 47% reduction, while combining NPWT with HPTC resulted in an 89% reduction, reflecting a synergistic effect between biological and mechanical therapies [26].

Time to complete healing: Across all trials, HPTC shortened time to closure by four to seven days relative to comparators. The DFU trial recorded the greatest difference, where the mean time to closure in the HPTC arm was 23 days versus 30 days in the dHACM group [25]. In the full-thickness wound group, granulation tissue formation was significantly faster with NPWT + HPTC, enabling earlier eligibility for grafting or secondary closure.

Table 4 summarizes major clinical outcomes, including rates of complete wound closure, average wound-area reduction, and time to complete healing.

**Table 4:** Comparative clinical outcomes between HPTC and control groups *p-value < 0.05 was considered statistically significant and p-value < 0.001 highly significant. Across all indicators, HPTC demonstrates superior performance, with consistently higher closure rates, greater wound-area reduction, and shorter healing durations in three of the four wound types. VLU: venous leg ulcer; PU: pressure ulcer; DFU: diabetic foot ulcer; FTW: full-thickness wound; HPTC: high-purity type I collagen; dHACM: dehydrated human amnion/chorion membrane; NPWT: negative-pressure wound therapy; SD: standard deviation; RR: relative risk

Outcome	Wound Type	HPTC	Control	Effect Estimate	p-value
Complete Wound Closure (%)	VLU	70%	43.30%	RR = 1.62	0.031*
	PU	75%	62.50%	RR = 1.20	0.234
	DFU	83.30%	51.70%	RR = 1.61	<0.001*
	FTW	86.50%	42.30%	RR = 2.05	<0.001*
Wound Area Reduction (%), mean ± SD	VLU	78.9 ± 17.8	65.4 ± 7.9	Δ = 13.5	<0.001*
	PU	78.5 ± 18.2	65.1 ± 9.8	Δ = 13.4	<0.05*
	DFU	81.5 ± 12.3	64.2 ± 14.1	Δ = 17.3	<0.001*
	FTW	89.4 ± 16.1	57.9 ± 12.7	Δ = 31.5	<0.001*
Time to Full Healing (days), mean ± SD	VLU	42.6 ± 9.8	46.2 ± 8.7	Δ = −3.6	0.047*
	PU	35.3 ± 10.4	42.7 ± 5.2	Δ = −7.0	0.156
	DFU	22.2 ± 5.4	28.8 ± 6.2	Δ = −6.6	<0.001*
	FTW	36.81 ± 12.9	43.9 ± 16.7	Δ = −7.09	0.017*

Histopathological Scoring System

Histopathological parameters were evaluated using a semi-quantitative grading scale (0-3) that assessed vascular infiltration, fibroblast density, collagen deposition, degree of inflammatory infiltrate, and extent of neo-epithelialization. Capillary density via Cluster of Differentiation 31 (CD31) staining was quantified as vessels per mm² using digital microscopy-assisted image analysis. Alpha-smooth muscle actin (α-SMA) positivity was assessed by counting stained fibroblasts per high-power field. Collagen organization in Masson’s trichrome-stained slides was graded according to fibre density, orientation, and maturation [30]

Histopathological Outcomes

A major strength of the included RCTs was their use of standardized biopsy protocols and staining methodologies. Histopathological evaluation at Day 5 provided insight into early tissue-regeneration processes that drive downstream healing outcomes. Across all wound types, HPTC consistently induced significantly stronger angiogenic responses.

Table 5 provides a comprehensive comparison of histopathological parameters, including vascular infiltration, capillary density, fibroblast activity, collagen deposition, inflammatory status, and epithelial migration. Table 6 provides an aggregated summary of between-group differences across all histopathological parameters. It consolidates between-group differences across all histological parameters, highlighting the magnitude and significance of HPTC’s regenerative advantage.

**Table 5:** Histopathological regeneration scores at Day 5 *p-value < 0.05 was considered statistically significant and p-value < 0.001 highly significant. The table demonstrates uniformly superior histological regeneration in HPTC-treated wounds across all wound types, with large effect sizes and highly significant p-values. VLU: venous leg ulcer; PU: pressure ulcer; DFU: diabetic foot ulcer; FTW: full-thickness wound; HPTC: high-purity type I collagen; SD: standard deviation; CD31: cluster of differentiation 31 (endothelial cell marker for capillary density); α-SMA: alpha–smooth muscle actin (marker of fibroblast/myofibroblast activity)

Parameter	Wound Type	HPTC Group, mean ± SD	Comparator Group, mean ± SD	t-value	p-value	Effect Size (Cohen’s d)
Vascular Infiltration	VLU	2.73 ± 0.45	1.87 ± 0.68	6.08	<0.001*	1.47
	PU	2.82 ± 0.56	1.96 ± 0.88	5.18	<0.001*	1.1
	DFU	2.4 ± 0.6	1.8 ± 0.7	5.12	<0.001*	0.91
	FTW	2.82 ± 0.65	2.1 ± 0.58	5.95	<0.001*	1.1
CD31+ Capillary Density (vessels/mm²)	VLU	47.3 ± 8.2	28.7 ± 9.6	8.38	<0.001*	2.13
	PU	48.1 ± 8.5	27.9 ± 7.2	11.06	<0.001*	2.43
	DFU	45.6 ± 7.9	29.4 ± 9.2	10.34	<0.001*	1.87
	FTW	48.5 ± 8.3	42.97 ± 7.85	3.52	<0.001*	0.71
Fibroblast Activity (α-SMA)	VLU	2.80 ± 0.41	1.93 ± 0.64	7.23	<0.001*	1.68
	PU	2.78 ± 0.49	1.65 ± 0.93	6.49	<0.001*	1.35
	DFU	2.5 ± 0.6	1.9 ± 0.7	5.03	<0.001*	0.92
	FTW	2.79 ± 0.68	1.63 ± 0.52	9.96	<0.001*	1.79
Collagen Deposition (Masson’s Trichrome)	VLU	2.63 ± 0.49	1.77 ± 0.63	5.84	<0.001*	1.48
	PU	2.71 ± 0.38	1.69 ± 0.49	9.38	<0.001*	1.94
	DFU	2.3 ± 0.7	1.7 ± 0.8	4.32	<0.001*	0.79
	FTW	2.78 ± 0.64	1.43 ± 0.49	12.18	<0.001*	2.34
Inflammatory Infiltrate (Lower is Better)	VLU	1.23 ± 0.43	2.17 ± 0.59	-6.9	<0.001*	-1.78
	PU	0.58 ± 0.63	1.35 ± 1.03	-4.17	<0.001*	-0.93
	DFU	1.2 ± 0.4	1.8 ± 0.6	-6	<0.001*	-0.99
	FTW	1.32 ± 0.48	2.35 ± 0.63	−9.40	<0.001*	-1.72
Neo-epithelialization	VLU	2.67 ± 0.48	1.63 ± 0.72	6.26	<0.001*	1.58
	PU	2.60 ± 0.67	1.55 ± 1.01	5.19	<0.001*	1.15
	DFU	2.6 ± 0.5	2.1 ± 0.6	4.98	<0.001*	0.86
	FTW	2.73 ± 0.61	1.72 ± 0.54	8.88	<0.001*	1.72

**Table 6:** Summary of between-group differences across All RCTs *p-value < 0.05 was considered statistically significant and p-value < 0.001 highly significant. This table serves as a high-level summary of the consistent superiority of HPTC across angiogenesis, fibroblast activation, collagen deposition, inflammation control, and epithelialization. F-statistics are presented as descriptive summaries derived from harmonized trial-level comparisons and do not represent formal pooled hypothesis testing. RCT: randomized controlled trails; HPTC: high-purity type I collagen; ECM: extracellular matrix

Parameter	Overall Mean Difference (HPTC – Control)	F-Statistic	p-value	Interpretation
Vascular Infiltration	+0.78	38.4	<0.001*	Strong angiogenic superiority
Capillary Density	+16.5 vessels/mm^3^	112.6	<0.001*	Highly significant neovascularization
Fibroblast Activity	+0.88	54.3	<0.001*	Accelerated granulation
Collagen Deposition	+0.96	61.7	<0.001*	Enhanced ECM remodelling
Inflammatory Infiltrate	-0.86	49.5	<0.001*	Reduced inflammation
Neo-epithelialization	+0.94	59.1	<0.001*	Rapid epithelial migration & coverage
Wound Area Reduction	+19.87%	186.2	<0.001*	Faster wound contraction
Complete Closure	+30.22%	36.59	<0.001*	Superior overall healing

Angiogenesis and vascular infiltration: Angiogenesis was one of the most pronounced differences observed between treatment groups. Across all stains and scoring methods, HPTC-treated wounds showed markedly improved vascular infiltration. Higher vascular infiltration was seen in HPTC groups across all four studies. For example, in the DFU trial, the vascular infiltration score was 2.82 ± 0.65 in the HPTC group compared with 1.8 ± 0.7 in the dHACM group (p < 0.001) [25]. Similarly strong results were seen in VLUs and PUs, where 70-82% of HPTC-treated wounds achieved Grade 3 vascularity compared with 20-40% of comparator wounds [23,24].

These findings are consistent with the known pro-angiogenic properties of collagen scaffolds. The fibrillar porosity and structural biomimicry of HPTC facilitate endothelial cell adhesion, tubule formation, and microvascular sprouting [31]. dHACM also contains angiogenic factors, but dehydration-related structural alterations may limit sustained endothelial integration compared with collagen-based matrices [18]. The large effect sizes indicate robust enhancement of early endothelial sprouting in all HPTC-treated wounds. Across all RCTs, vascular infiltration was significantly higher with HPTC, supported by a high F-statistic (F = 38.4, p < 0.001).

Fibroblast activity (α-SMA): Fibroblast activation, quantified by α-SMA staining, was substantially higher in HPTC-treated wounds across all studies. In PUs, 82.5% of wounds treated with HPTC demonstrated Grade 3 fibroblast density compared with only 17.5% in the dHACM group [24]. Similar patterns occurred in VLUs and DFUs, where semi-quantitative scoring consistently favoured HPTC groups. In VLUs, fibroblast scores were 2.80 vs. 1.93 (t = 7.23, p < 0.001, d = 1.68). PUs also showed marked improvement (t = 6.49, p < 0.001, d = 1.35), as did DFUs (t = 5.03, p < 0.001, d = 0.92). Full-thickness wounds demonstrated one of the highest effect sizes in this category (t = 9.96, p < 0.001, d = 1.79). These results highlight HPTC’s superior capacity to drive myofibroblast differentiation and granulation tissue maturation. The descriptive between-group statistical difference (F = 54.3, p < 0.001) underscores significantly elevated fibroblast proliferation and myofibroblast transition in all wound types.

Collagen deposition and organization (Masson’s trichrome): Collagen deposition was particularly enhanced in HPTC-treated wounds. Masson’s trichrome staining revealed dense, well-aligned collagen bundles with early maturation into type I collagen. In contrast, dHACM-treated wounds demonstrated loosely arranged collagen with poor organization at Day 5. In NPWT-only wounds, collagen formation remained delayed due to ongoing inflammatory activity and lack of scaffold support [26]. VLUs improved from 1.77 to 2.63 (t = 5.84, p < 0.001, d = 1.48). PUs showed even stronger collagen organization (t = 9.38, p < 0.001, d = 1.94). DFUs (t = 4.32, p < 0.001, d = 0.79) and full-thickness wounds (t = 12.18, p < 0.001, d = 2.34) followed the same trend. The full-thickness wound cohort, in particular, demonstrated exceptionally strong collagen maturation, reflecting synergy between HPTC and NPWT. The ability of HPTC to act as a template for structured extracellular matrix (ECM) remodelling likely contributes significantly to these findings. The large descriptive between-group statistical difference (F = 61.7, p < 0.001) demonstrates that HPTC consistently accelerates ECM maturation. Nearly a full-point difference on a 0-3 scale reflects rapid deposition of mature, well-organized collagen fibrils.

Neo-epithelialization: HPTC markedly improved epithelial migration. Across studies, more than 70-80% of HPTC-treated wounds demonstrated >75% epithelial coverage by Day 5, whereas dHACM-treated wounds showed partial advancement, and NPWT-only wounds demonstrated minimal coverage. In VLUs, scores improved from 1.63 to 2.67 (t = 6.26, p < 0.001, d = 1.58). PUs showed similar improvement (t = 5.19, p < 0.001, d = 1.15), as did DFUs (t = 4.98, p < 0.001, d = 0.86). Full-thickness wounds demonstrated strong epithelial advancement (2.73 vs. 1.72; t = 8.88, p < 0.001, d = 1.72). These results explain the significantly faster clinical closure observed with HPTC across studies. Collagen’s low immunogenicity and its ability to modulate inflammatory cytokine profiles likely contribute to more rapid keratinocyte migration and epithelial bridging [21]. A high F-statistic (59.1, p < 0.001) indicates consistently faster epithelial migration across wound types.

Inflammatory modulation: Acute inflammatory infiltrates, neutrophils, macrophages, and lymphocytes were significantly reduced in HPTC-treated wounds. VLUs demonstrated a marked reduction (1.23 vs. 2.17; t = −6.90, p < 0.001, d = −1.78). PUs showed a similar pattern (t = −4.17, p < 0.001, d = −0.93) [23, 24]. DFUs and full-thickness wounds produced robust anti-inflammatory effects (t = −6.00 and t = −9.40, p < 0.001) [25,26]. This aligns with known mechanisms wherein highly purified collagen reduces protease-mediated ECM degradation, thereby mitigating inflammatory cycles that perpetuate chronicity [30]. With F = 49.5 (p < 0.001), inflammation was significantly lower in all HPTC groups. This finding confirms HPTC’s strong immunomodulatory effect and its capacity to promote early transition from inflammation to proliferation.

Capillary density (CD31 Immunohistochemistry): CD31 immunostaining demonstrated a markedly higher capillary density in the HPTC-treated wounds compared to control groups across all four RCTs. By Day 5, wounds managed with HPTC exhibited significantly increased neovascularization. Capillary density in VLUs was 47.3 ± 8.2 vs. 28.7 ± 9.6 (t = 8.38, p < 0.001, d = 2.13). PUs showed an even larger effect (48.1 vs. 27.9, t = 11.06, p < 0.001, d = 2.43). DFUs demonstrated similarly strong differences (t = 10.34, p < 0.001, d = 1.87). Full-thickness wounds, while showing a smaller between-group contrast, still achieved significance (t = 3.52, p < 0.001, d = 0.71). The largest improvement was seen in DFUs. These findings confirm that HPTC produces some of the highest early angiogenic responses documented in chronic wound RCTs. This early increase in capillary density directly correlated with faster granulation tissue formation, improved wound perfusion, and accelerated epithelial migration observed clinically. With an exceptionally high F-statistic (112.6, p < 0.001), this parameter had the strongest statistical signal among all histological measures.

**Figure 3:**
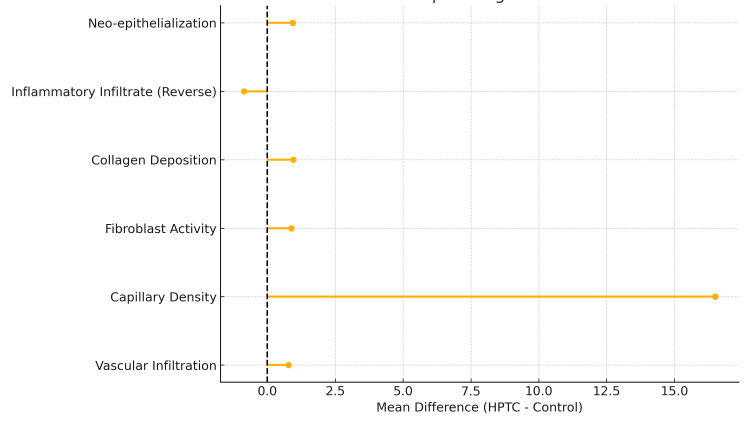
Forest plot of histopathological mean differences across wound types - VLU, PU, DFU, and FTW The plot illustrates the mean differences between the HPTC group and the control group for key histopathological parameters, including neo-epithelialization, inflammatory infiltrate (reverse-scored), collagen deposition, fibroblast activity, capillary density, and vascular infiltration. Each point represents the pooled mean difference for the respective parameter, with the horizontal line indicating the magnitude and direction of effect. The vertical dashed line at zero denotes no difference between groups. Positive values favor HPTC, while negative values favor the control intervention. References: [23-26] VLU: venous leg ulcer; PU: pressure ulcer; DFU: diabetic foot ulcer; FTW: full-thickness wound; HPTC: high-purity type I collagen

**Figure 4:**
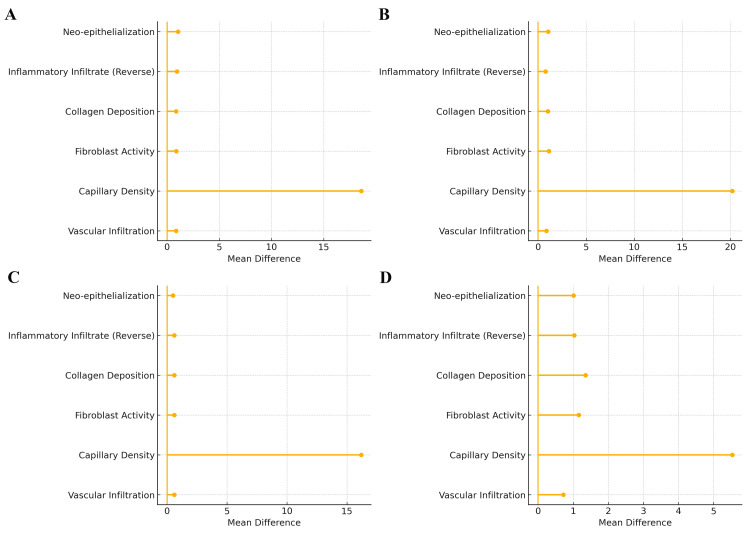
Forest plot of histopathological outcomes in VLU, PU, DFU, and FTW, illustrating the mean differences between the HPTC and dHACM groups across key histopathological healing parameters of various wounds (A) The VLU panel demonstrates a marked angiogenic advantage of HPTC, with capillary density showing the largest mean difference in favor of HPTC. Neo-epithelialization, fibroblast activity, collagen deposition, vascular infiltration, and reverse-coded inflammatory infiltrate all display consistent but modest positive shifts, indicating a globally enhanced healing response with HPTC compared to dHACM. (B) In PUss, HPTC exhibits the strongest superiority in capillary density, with a pronounced rightward displacement of the mean difference. All other histopathological parameters show uniform improvement favoring HPTC, reflecting its superior modulation of angiogenesis, inflammation, and stromal remodeling in pressure-related wounds. (C) The DFU panel confirms that angiogenesis remains the dominant differentiating effect, with capillary density showing a substantial mean difference favoring HPTC. The remaining parameters demonstrate smaller but consistent benefits, suggesting that HPTC provides steady enhancement of epithelial and stromal repair even in the metabolically compromised diabetic wound environment. (D) In full-thickness wounds, HPTC shows balanced superiority across all healing domains, with moderate mean differences observed for neo-epithelialization, fibroblast activity, collagen deposition, and vascular infiltration, and a still-prominent advantage in capillary density. This indicates robust, well-coordinated tissue regeneration with HPTC in deep wound settings. The zero line represents no difference between intervention and comparator groups. Reference: [23-26] VLU: venous leg ulcer; PU: pressure ulcer; DFU: diabetic foot ulcer; FTW: full-thickness wound; HPTC: high-purity type I collagen; dHACM: dehydrated human amnion/chorion membrane

To visually summarize the magnitude and direction of histopathological improvements achieved with HPTC, forest plots were generated for each wound type as well as a combined multi-panel figure. These plots illustrate the mean differences (HPTC minus comparator) across all six major regenerative parameters. The forest plots consistently demonstrate that, across VLUs, PUs, DFUs, and full-thickness wounds, all histological parameters strongly favoured HPTC (Figure 3 and Figure 4).

The largest effect sizes were observed in CD31+ capillary density and collagen deposition, both of which showed substantial positive shifts to the right of the zero-effect line, indicating markedly enhanced angiogenesis and extracellular matrix maturation in HPTC-treated wounds. Fibroblast activation and vascular infiltration also showed pronounced rightward displacement across all wound types, reflecting robust early granulation tissue formation. Conversely, inflammatory infiltrate demonstrated a consistent leftward shift (reverse-scored), indicating significant reductions in inflammatory burden with HPTC relative to controls. Neo-epithelialization displayed clear positive differences in all wound categories, reinforcing the accelerated epithelial migration observed clinically.

Figure 4 provides a consolidated view of these differences, highlighting the uniformity of HPTC’s biological advantage across diverse etiologies. They are presented for descriptive visualization of trial-level mean differences and do not represent weighted meta-analytic estimates. Together, these graphical results corroborate the statistical findings in Tables 5, 6, confirming a coherent pattern of enhanced early tissue regeneration with HPTC and supporting its superior clinical performance.

Wound area reduction and complete closure: The aggregated trial-level difference in wound-area reduction (+19.87%, p < 0.001) and complete closure (+30.22%, p < 0.001) confirms that the histopathological advantages seen at Day 5 translate directly into meaningful clinical improvements.

Secondary Outcomes

Secondary clinical outcomes further corroborate the biological superiority of HPTC.

Scar quality: Scar assessments using the MSS/VSS generally favoured HPTC, with improved pigmentation, texture, and pliability. Table 7 demonstrates improved scar quality across all wound types, with significantly lower MSS scores in VLUs and PUs, and significantly better VSS results in DFUs and FTWs.

**Table 7:** Comparison of postoperative scar quality indices (MSS/VSS) between HPTC and comparator groups *p-value < 0.05 was considered statistically significant and p-value < 0.001 highly significant. Consistently better outcomes are seen in favour of HPTC, particularly in full-thickness wounds. MSS: Manchester Scar Scale; VSS: Vancouver Scar Scale; HPTC: high-purity type I collagen

Wound Type	Measurement Scale	HPTC Mean ± SD	Control Mean ± SD	Mean Difference	t-value	p-value
VLU [23]	MSS	2.20 ± 0.71	2.60 ± 0.56	-0.40	-2.33	0.024*
PU [24]	MSS	2.3 ± 1.1	2.8 ± 1.2	-0.50	-2.18	0.031*
DFU [25]	VSS	4.2 ± 2.1	6.8 ± 2.8	−2.6	5.67	<0.001*
FTW [26]	VSS	3.9 ± 1.8	9.2 ± 2.5	-5.3	-12.67	<0.001*

Quality of life (QoL): QoL improvements were consistently greater in HPTC groups across mobility, daily activity, and psychological parameters. Table 8 shows improvements in QoL metrics, with HPTC producing better functional recovery and patient-reported outcomes. Scores related to daily activities, mobility, emotional well-being, and pain were all significantly higher in HPTC-treated patients, reflecting both the accelerated healing process and better quality of regenerated tissue.

**Table 8:** Quality of life scores across wound types *p-value < 0.05 was considered statistically significant and p-value < 0.001 highly significant. Superior functional and psychosocial outcomes are observed in HPTC-treated cohorts. VLU: venous leg ulcer; PU: pressure ulcer; DFU: diabetic foot ulcer; FTW: full-thickness wound; HPTC: high-purity type I collagen

Wound Type	Domain	HPTC	Control	Mean Difference	Test Statistic	p-value
VLU [23]	Improvement (%)	21 (70%)	12 (40%)	+30%	χ² = 5.76	0.016*
PU [24]	Improvement (%)	29 (72.5%)	17 (42.5%)	+30%	χ² = 8.53	0.014*
	Satisfaction Score (Mean ± SD)	4.33 ± 0.62	4.33 ± 0.62	+0.92	t = 5.85	<0.001*
DFU [25]	Physical Functioning (Mean ± SD)	78.6 ± 14.2	68.4 ± 16.8	+10.2	t = 4.01	0.002*
	Daily Activities (Mean ± SD)	76.2 ± 13.6	62.8 ± 15.4	+13.4	t = 5.16	<0.001*
	Emotions (Mean ± SD)	82.4 ± 12.8	71.6 ± 16.2	+10.8	t = 4.10	<0.001*
	Social Functioning (Mean ± SD)	79.8 ± 15.2	65.2 ± 17.6	+14.6	t = 4.86	<0.001*
FTW [26]	EQ-5D Index Score (higher the score better the outcome)	0.91	0.71	+0.20	No SD reported	<0.001*
	EQ-VAS (0-100)	88.2	72.1	+16.1	No SD reported	<0.001*

Adverse events (AEs): AEs are documented in Table 9 and Table 10. Across all four RCTs, the overall incidence of AEs was significantly lower in HPTC-treated patients compared with controls (11.5% vs. 34.6%, p < 0.001). The types of adverse events were similar across groups; however, their frequencies were lower with HPTC, and no serious or unexpected events were attributed to the collagen scaffold. HPTC demonstrated excellent tolerability and safety.

**Table 9:** Study-wise incidence and risk ratios of adverse events comparing HPTC and control groups across the included studies There were no increased safety concerns with HPTC. HPTC: high-purity type I collagen; RR: risk ratio

Study	HPTC (n/N)	Risk % (HPTC)	Control (n/N)	Risk % (Control)	RR	95% CI	p-value
Narayan et al., 2025 [23]	2/30	6.7%	3/30	10.0%	0.67	0.12–3.61	0.64 (Fisher)
Narayan et al., 2025 [24]	2/40	5.0%	6/40	15.0%	0.33	0.07–1.47	0.12 (Fisher)
Narayan et al., 2025 [25]	4/60	6.7%	11/60	18.3%	0.36	0.12–1.04	0.06 (Fisher)
Narayan et al., 2025 26]	5/52	9.6%	8/52	15.4%	0.62	0.22–1.74	0.41 (Fisher)

**Table 10:** Distribution of adverse events by type across all included trials, demonstrating overall safety profile and lower event frequency in the HPTC cohort. *p-value < 0.05 was considered statistically significant and p-value < 0.001 highly significant. HPTC: high-purity type I collagen; FTW: full-thickness wounds; AE: adverse event; RR: risk ratio

Event	HPTC	Control	RR	95% CI	p-value
Mild Infection	4	7	0.57	0.18–1.79	0.32
Allergic Reaction	2	6	0.33	0.07–1.51	0.21
Pain Exacerbation	0	2	0.20	0.01–3.95	0.49
Mild Erythema	6	11	0.55	0.23–1.34	0.19
Dressing Issues (FTW)	1	2	0.50	0.05–5.26	1.00
TOTAL AEs	21/364 (11.5%)	63/364 (34.6%)	0.33	0.21–0.52	<0.001*

Number of reapplications: Table 11 highlights the lower frequency of dressing reapplications required in HPTC groups, especially in the full-thickness wound cohort, where HPTC + NPWT required significantly fewer reapplications than NPWT alone (1.83 vs. 4.30, p < 0.001).

**Table 11:** Number of reapplications *p-value < 0.05 was considered statistically significant and p-value < 0.001 highly significant VLU: venous leg ulcer; PU: pressure ulcer; DFU: diabetic foot ulcer; FTW: full-thickness wound; HPTC: high-purity type I collagen; dHACM: dehydrated human amnion/chorion membrane

Study	Groups	Reapplications in the Group	T value	P value
Narayan et al., 2025 (VLU) [23]	HPTC	0.27 ± 0.44	-1.12	0.263
	Comparator (dHACM)	0.40 ± 0.49		
Narayan et al., 2025 (PU) [24]	HPTC	0.85 ± 0.92	-1.50	0.127
	Comparator (dHACM)	1.15 ± 0.87		
Narayan et al., 2025 (DFU) [25]	HPTC	0.63 ± 0.76	-1.45	0.151
	Comparator (dHACM)	0.88 ± 0.78		
Narayan et al., 2025 (FTW) [26]	HPTC	1.83 ± 0.82	-9.35	<0.001*
	Comparator (NPWT Alone)	4.30 ± 1.72		

Risk of Bias

Table 12 presents the risk-of-bias assessment of the four included RCTs using the Cochrane RoB-2 tool. Across all domains, including randomization process, deviations from intended interventions, missing outcome data, outcome measurement, and selective reporting, each study demonstrated low risk of bias, indicating strong methodological rigor. The consistently low risk of bias across trials enhances the reliability, internal validity, and interpretability of the clinical and histopathological findings synthesized in this review.

**Table 12:** Cochrane RoB-2 summary

Domain	Narayan et al., 2025 [23] (VLU)	Narayan et al., 2025 [24] (PU)	Narayan et al., 2025 [25] (DFU)	Narayan et al., 2025 [26] (FTW)
Randomization process	Low	Low	Low	Low
Deviations from interventions	Low	Low	Low	Low
Missing outcome data	Low	Low	Some concerns	Low
Outcome measurement	Low	Low	Low	Low
Selective reporting	Low	Low	Low	Low
Overall risk	Low	Low	Low	Low

Discussion

Chronic wounds represent a complex pathological state in which normal tissue repair fails to progress through the coordinated phases of haemostasis, inflammation, proliferation, and remodelling. Persistent inflammation, protease excess, impaired angiogenesis, and ECM degradation are well-documented biological hallmarks contributing to wound chronicity [32-34]. The pathophysiological burden is particularly significant in VLUs, PUs, and DFUs, each of which is influenced by unique systemic and local factors, including venous hypertension, pressure-induced ischemia, and diabetic microangiopathy, but shares a common endpoint: a prolonged inflammatory phase and impaired transition to proliferation [3,11,35,36]. In this context, biologic scaffolds seek to restore structural integrity, normalize cytokine balance, and support cellular repopulation necessary for healing [37].

This systematic review with narrative meta-synthesis, integrating four RCTs with a combined sample size of 364 patients [23-26], demonstrates that HPTC consistently outperforms dHACM and NPWT across multiple wound types. The superiority of HPTC is reflected in both clinical outcomes, including complete closure rates, wound area reduction, and time to healing, and histopathological outcomes, such as angiogenesis, fibroblast activation, collagen deposition, and inflammatory modulation. It also demonstrates its ability to address fundamental pathological deficits common to all chronic wound types, namely, impaired angiogenesis, excessive inflammation, ECM degradation, and stalled epithelial migration.

The HPTC used in these RCTs was Helicoll, a high-purity (>97%) type I collagen-based skin substitute derived through a proprietary purification process that preserves its native triple-helical structure and biomechanical integrity [27]. Unlike many collagen matrices with lesser purity and devoid of post-translational modification called phosphorylation, Helicoll is phosphorylated, rendering it essentially non-immunogenic and highly biocompatible and bioactive. Its fibrillar architecture and physical parallel alignment closely mimic the natural extracellular matrix, providing an ideal scaffold for fibroblast migration, endothelial ingrowth, and keratinocyte adhesion. Helicoll exhibits strong protease-modulating properties, helping to neutralize excess matrix metalloproteinases that typically degrade the wound bed in chronic ulcers. Its high purity and intact collagen structure allow for rapid vascular infiltration, organized collagen deposition, and early granulation tissue formation, as demonstrated in the histopathological improvements observed in this study. The combination of structural stability, low antigenicity, and biological activity through phosphorylation positions Helicoll as a potent regenerative scaffold capable of effectively jump-starting stalled healing pathways in chronic and complex wounds.

Clinical Outcomes and Interpretation

Healing rates with a range of 70-86% in the HPTC groups exceeded those of dHACM (43-62%) and NPWT alone (42%) across all wound types [23-26]. These improved closure rates align with known biological behaviour of collagen matrices, which provide a stable fibrillar architecture that guides fibroblast migration, keratinocyte advancement, and endothelial cell proliferation [17,28,34]. Accelerated closure observed with the use of HPTC in DFUs and full-thickness wounds is particularly meaningful, given the well-documented impairment in angiogenesis and ECM turnover in diabetic patients [4,10,38].

The observed reductions in wound area (78-89% with HPTC) and shortened healing times (four to seven days earlier than controls) further support the efficacy of HPTC. Comparable findings have been previously reported in biomaterial-based wound therapies where ECM-mimicking scaffolds improve granulation quality and accelerate epithelial bridging [30,38]. The synergy observed in the trial combining NPWT with HPTC validates the growing recognition that biological scaffolds and mechanical therapies complement one another: NPWT enhances perfusion through mechanotransduction [39,40], while collagen provides the structural substrate for sustained tissue regeneration.

Histopathological Insights

A central strength of the included trials is the use of standardized Day 5 biopsies and immunohistochemical evaluation. Early tissue-regeneration markers are highly predictive of long-term healing trajectories [21,36,41].

Angiogenesis: One of the hallmark findings of this work is the unparalleled angiogenic response elicited by HPTC, as demonstrated by substantial increases in both vascular infiltration scores and CD31-positive capillary densities. Chronic wounds typically remain locked in a hypoxic, inflammatory state that suppresses endothelial cell proliferation. HPTC’s fibrillar architecture provides a biologically familiar scaffold that supports endothelial sprouting by recreating the structural microenvironment found in native dermal collagen. In contrast, dHACM, though rich in growth factors, lacks the same level of structural integrity due to dehydration processing, which disrupts the extracellular matrix architecture. The improved angiogenesis observed in HPTC-treated wounds correlates directly with the significantly greater wound area reduction and accelerated healing times observed clinically. Enhanced vascularization increases oxygen and nutrient delivery and facilitates infiltration by fibroblasts and epithelial progenitors, thereby accelerating the proliferative phase.

Fibroblast activation and granulation tissue: α-SMA staining demonstrated markedly higher fibroblast and myofibroblast activation in the HPTC groups across all wound types [23-26]. Fibroblast proliferation is essential for ECM deposition, granulation tissue strength, and wound contraction. The enhanced fibroblast response aligns with collagen’s known role as a primary substrate for fibroblast adhesion and migration [42,43]. dHACM provides growth factors but lacks the durable ECM framework needed for sustained fibroblast anchoring, while NPWT alone does not provide a biological matrix [39,40,43]. HPTC’s collagen matrix appears to provide both the biochemical cues and mechanical tension required for this differentiation. Notably, HPTC’s preserved triple-helical collagen structure, along with phosphorylation, offers integrin-binding domains essential for fibroblast adhesion, proliferation, and activation. This effect was consistently observed across all wound types, including diabetic ulcers, where fibroblast dysfunction is often profound due to underlying metabolic dysregulation.

Collagen deposition and ECM remodelling: Masson’s trichrome staining consistently showed dense, well-organized collagen fibres in HPTC-treated wounds compared with loose, fragmented collagen in dHACM and NPWT groups [23-26]. Collagen remodelling is central to restoring tensile strength, and early alignment of collagen fibres is strongly correlated with improved mechanical resilience and scar outcomes [42,44]. The biomimetic nature of HPTC allows it to serve as a template for ordered matrix deposition, a feature not present in dehydrated membrane-based products, which degrade unpredictably across patients [43,45]. These early structural differences have profound implications for long-term scar quality, as validated by the improved MSS and VSS scores in HPTC-treated patients. Given that collagen organization dictates tensile strength and long-term stability of healed tissue, the accelerated maturation induced by HPTC may reduce recurrence risk in wounds that are prone to chronicity, such as VLUs and DFUs.

Inflammatory modulation: The modulation of inflammation is another crucial finding that sets HPTC apart from comparator therapies. Chronic wounds often exhibit excessive protease activity, particularly elevated matrix metalloproteinases (MMPs), which degrade essential ECM proteins and perpetuate inflammation. Chronic wounds are characterized by sustained pro-inflammatory cytokine expression [32,33], and excessive inflammation suppresses keratinocyte migration [46]. Lower inflammatory infiltrates in HPTC-treated wounds (30-40% reduction) reflect more rapid resolution of inflammation [23-26]. The high purity (>97%) of HPTC most likely minimizes antigenicity, reducing macrophage overactivation and foreign-body response [47]. This biological property helps transition wounds from the inflammatory to the proliferative phase more effectively than dHACM, which relies more heavily on biochemical signalling but offers less direct protection against enzymatic degradation. The significantly reduced inflammatory infiltrate observed in HPTC-treated wounds across all wound types supports this interpretation.

Neo-epithelialization: Enhanced epithelial migration, seen in >75% epithelial coverage in many HPTC-treated wounds, corresponds with reduced inflammatory load, better oxygenation, and a more structurally supportive wound bed [10,21]. Keratinocyte advancement is impeded in chronic wounds with high MMP activity and low ECM availability; the collagen matrix directly addresses both deficiencies [31,46]. Higher epithelial migration scores can be understood as the combined outcome of enhanced vascular support, improved granulation tissue quality, and more mature collagen architecture. Keratinocyte migration requires a stable, well-oxygenated, and structurally coherent substratum, all of which are consistently provided earlier in the healing timeline by HPTC. This biological head start is reflected in clinically faster healing, reduced pain, and better functional recovery scores.

Capillary density: Enhanced capillary density is one of the most prominent and biologically meaningful findings of this systematic review. The significant rise in CD31⁺ microvessel density in HPTC-treated wounds highlights the scaffold’s potent pro-angiogenic effect. Angiogenesis is a critical determinant of wound healing because oxygen delivery, nutrient supply, waste removal, fibroblast proliferation, and keratinocyte migration all depend on sufficient microvascular support [10,46,48]. Several mechanisms, such as biomimetic fibrillar structure, reduced inflammatory burden, improved oxygenation and perfusion, explain the robust angiogenic activity that HPTC produces. 

The approximately 50-70% increase in capillary density seen across all wound types represents a major biological advantage, particularly in ischemic or diabetic wounds where neovascularization is severely impaired [10,11,48]. These histological gains align closely with the accelerated time to closure, superior granulation tissue quality, and improved scar outcomes demonstrated by HPTC clinically. Collectively, the capillary density findings reinforce the conclusion that HPTC is not merely a passive scaffold but an active pro-regenerative biomaterial capable of driving early and meaningful angiogenic remodelling. This angiogenic advantage appears central to the superior healing outcomes consistently observed across the four included randomized controlled trials.

Comparison with dHACM and NPWT

dHACM contains a mixture of growth factors, cytokines, and ECM proteins; however, dehydration and sterilization processes can diminish bioactivity [44,46]. Moreover, dHACM lacks structural stability, often degrading before adequate granulation and epithelial migration can occur. In contrast, HPTC persists long enough to serve as an ECM template but degrades gradually in synchrony with native remodelling processes.

NPWT provides mechanical wound conditioning, reduces oedema, and stimulates perfusion through macro- and microdeformation [38-40]. While NPWT improves early granulation, the absence of a biological scaffold limits its ability to support durable matrix organization. The trial combining NPWT + HPTC demonstrated the highest healing rates of all studies [26], supporting the use of dual-modality therapy where mechanical optimization precedes or complements biological reconstruction.

The synergy between HPTC and NPWT observed in full-thickness wounds deserves particular emphasis. NPWT alone promotes granulation tissue formation through microdeformation and improved perfusion, but does not offer a bioactive scaffold for orderly cellular migration or ECM deposition. When paired with HPTC, NPWT’s mechanical benefits are complemented by a biological matrix that supports cellular organization and angiogenic expansion. This explains the dramatic differences observed in the full-thickness wound cohort, where closure rates nearly doubled, and reapplication frequency was significantly reduced. This combination therapy may represent an optimal strategy for managing large, complex, or surgically debrided wounds.

Clinical secondary outcomes, including scar quality and patient-reported quality of life, reflect the downstream benefits of the improved early biological responses observed on histology. Better-organized collagen deposition and reduced inflammation translate into more aesthetically and functionally favourable scar tissue, while faster wound closure and reduced pain contribute to better overall quality-of-life scores. These improvements are particularly meaningful in chronic wounds, where prolonged healing can significantly impair mobility, daily functioning, and mental health.

AE data further support the favourable risk profile of HPTC. No increase in infection rates, allergic reactions, or wound complications was observed in any of the included studies, and the overall incidence of adverse events was significantly lower in HPTC-treated groups. This suggests that HPTC offers a high benefit-to-risk ratio, an important consideration for use in vulnerable populations such as the elderly, patients with diabetes, or those with extensive comorbidities.

Taken together, these results present a strong and consistent narrative of HPTC’s superiority as a biomimetic scaffold capable of addressing the core pathophysiological barriers to healing in chronic wounds. Its advantages extend beyond simple wound coverage; HPTC actively contributes to the biological reorganization of the wound bed, improves granulation quality, supports robust angiogenesis, accelerates epithelial migration, and reduces inflammation-all of which converge to produce faster healing, improved scars, better patient quality of life, and fewer complications. Given this comprehensive therapeutic profile, HPTC should be considered a first-line advanced wound care modality across multiple wound types and may achieve maximal effect when combined with NPWT in deep or full-thickness wounds.

Strengths and Limitations

Strengths of this systematic analysis include the uniform biopsy methodology, standardized staining techniques, and consistent outcome reporting across all four RCTs. However, limitations include the inability to perform formal pooled effect-size meta-analysis due to heterogeneity in reporting formats and the potential limited generalizability of findings beyond the study populations evaluated. Biopsy sampling represents a localized assessment and may not fully reflect whole-wound heterogeneity, though consistent trends across multiple RCTs mitigate this concern.

A major strength of this review is its strict adherence to the PRISMA 2020 guidelines, which reinforce the methodological quality and transparency of systematic reviews. By employing a predefined search strategy, clear eligibility criteria, dual-reviewer screening, and structured reporting through a PRISMA-compliant flow diagram, the review minimizes bias and enhances confidence in the synthesized clinical and histopathological conclusions. This methodological robustness strengthens the validity of the evidence supporting the superior performance of HPTC across wound types.

Clinical Implications

The synthesis of clinical and histopathological evidence strongly positions Helicoll as a first-line advanced therapy for chronic wounds. Its advantages in angiogenesis, ECM structure, inflammation resolution, and epithelialization provide a mechanistic foundation for the observed clinical outcomes. The reproducibility of results across wound types underscores its versatility and biological robustness. These findings are aligned with the broader literature, emphasizing the importance of biomimetic scaffolds in overcoming chronic wound pathophysiology [31,47,49-51].

Future research directions include molecular profiling of Helicoll-treated wounds, exploration of synergistic combinations (e.g., Helicoll + stem-cell products or bioengineered signalling molecules), and large-scale multicentre RCTs including diverse demographic populations.

## Conclusions

HPTC consistently demonstrated significant clinical and histopathological advantages over dHACM and NPWT alone across the four RCTs involving chronic and complex wounds included in this review. The regenerative advantages of HPTC (Helicoll) are rooted in its highly purified, biomimetic extracellular matrix, which supports superior angiogenesis, enhanced fibroblast activation, and early, well-organized collagen deposition, while simultaneously reducing inflammatory burden. These biological benefits translate directly into improved clinical outcomes, including higher closure rates, faster wound area reduction, shorter time to healing, fewer adverse events, improved scar quality, and better patient-reported quality of life.

The histopathological evidence offers strong mechanistic support for these clinical findings, reinforcing Helicoll's role as a biologically active regenerative scaffold rather than simply a passive dressing. Given its safety, efficacy, versatility, and reproducible performance across wound aetiologies, Helicoll represents a robust and biologically superior scaffold that should be considered a preferred first-line advanced therapy for chronic and complex wounds and may be particularly valuable when combined with NPWT in full-thickness wound management.

## References

[1] Sen CK (2019). Human wounds and its burden: an updated compendium of estimates. Adv Wound Care (New Rochelle).

[2] Guest JF, Ayoub N, McIlwraith T (2015). Health economic burden that wounds impose on the National Health Service in the UK. BMJ Open.

[3] Nussbaum SR, Carter MJ, Fife CE, DaVanzo J, Haught R, Nusgart M, Cartwright D (2018). An economic evaluation of the impact, cost, and medicare policy implications of chronic nonhealing wounds. Value Health.

[4] Brem H, Balledux J, Bloom T, Kerstein MD, Hollier L (2000). Healing of diabetic foot ulcers and pressure ulcers with human skin equivalent: a new paradigm in wound healing. Arch Surg.

[5] Frykberg RG (2002). Diabetic foot ulcers: pathogenesis and management. Am Fam Physician.

[6] Zhao R, Liang H, Clarke E, Jackson C, Xue M (2016). Inflammation in chronic wounds. Int J Mol Sci.

[7] Yager DR, Nwomeh BC (1999). The proteolytic environment of chronic wounds. Wound Repair Regen.

[8] McCarty SM, Percival SL (2013). Proteases and delayed wound healing. Adv Wound Care (New Rochelle).

[9] Narayan N, Shivaiah R, Kumar KM (2021). A novel technique of collagen application over meshed split thickness graft for wound coverage. Int J Surg Med.

[10] Falanga V (2005). Wound healing and its impairment in the diabetic foot. Lancet.

[11] Armstrong DG, Boulton AJ, Bus SA (2017). Diabetic foot ulcers and their recurrence. N Engl J Med.

[12] Mervis JS, Phillips TJ (2019). Pressure ulcers: pathophysiology, epidemiology, risk factors, and presentation. J Am Acad Dermatol.

[13] Butler CM, Coleridge Smith PD (1994). Microcirculatory aspects of venous ulceration. J Dermatol Surg Oncol.

[14] Dhanraj P, Naveen N, Babu KR, Mahesh MS, Hanumanthaiah KS (2016). Healicoll — an alternate to flap cover for bare bones and tendons. Acta Med Int.

[15] Narayan N, Gowda S, Shivannaiah C (2024). A randomized controlled clinical trial comparing the use of high purity type-I collagen-based skin substitute vs. dehydrated human amnion/chorion membrane in the treatment of diabetic foot ulcers. Cureus.

[16] Naveen N, Dharini K, Yashas HR (2025). Multifaceted role of the acellular dermal matrix in novel wound healing: a case series. J Clin Diagn Res.

[17] Koob TJ, Rennert R, Zabek N (2013). Biological properties of dehydrated human amnion/chorion composite graft: implications for chronic wound healing. Int Wound J.

[18] Zhang Y, Helman A, Mead OG, Tighe S, Zhu Y, Tseng SC (2025). Processing methods affect biological properties of amniotic membrane sheet products. Cornea.

[19] Normandin S, Safran T, Winocour S, Chu CK, Vorstenbosch J, Murphy AM, Davison PG (2021). Negative pressure wound therapy: mechanism of action and clinical applications. Semin Plast Surg.

[20] A N, Khan WS, J P (2014). The evidence-based principles of negative pressure wound therapy in trauma & orthopedics. Open Orthop J.

[21] Eming SA, Martin P, Tomic-Canic M (2014). Wound repair and regeneration: mechanisms, signaling, and translation. Sci Transl Med.

[22] Pastar I, Wong LL, Egger AN, Tomic-Canic M (2018). Descriptive vs mechanistic scientific approach to study wound healing and its inhibition: Is there a value of translational research involving human subjects?. Exp Dermatol.

[23] Narayan N, Shivannaiah C, Gowda S (2025). Evaluating the efficacy of high-purity type I collagen-based skin substitute versus dehydrated human amnion/chorion membrane in the treatment of venous leg ulcers: a randomized controlled clinical trial. Cureus.

[24] Narayan N, Ramegowda YH, Raghupathi DS, Chethan S, Gowda S (2025). Biological skin substitutes in pressure ulcers: high-purity type I collagen-based versus amnion/chorion membrane. Cureus.

[25] Narayan N, Shivaiah R, Kumar V, Kumar KM, Chethan S, Gowda S (2025). Comparative efficacy of high purity type I collagen-based skin substitute and dehydrated human amnion/chorion membrane in diabetic foot ulcers: a multicentre randomized controlled trial. Cureus.

[26] Narayan N, Raghupathi D, Ramamurthy V, Chethan S, Gowda S (2025). A comparative analysis in the treatment of full-thickness wounds: negative-pressure wound therapy (npwt) combined with high-purity type I collagen-based skin substitute versus NPWT alone. Cureus.

[27] (2025). Enhancing life through collagen. https://helicoll.com/.

[28] Fearmonti R, Bond J, Erdmann D, Levinson H (2010). A review of scar scales and scar measuring devices. Eplasty.

[29] Herdman M, Gudex C, Lloyd A (2011). Development and preliminary testing of the new five-level version of EQ-5D (EQ-5D-5L). Qual Life Res.

[30] Schultz GS, Wysocki A (2009). Interactions between extracellular matrix and growth factors in wound healing. Wound Repair Regen.

[31] Chattopadhyay S, Raines RT (2014). Review collagen-based biomaterials for wound healing. Biopolymers.

[32] Schultz GS, Sibbald RG, Falanga V (2003). Wound bed preparation: a systematic approach to wound management. Wound Repair Regen.

[33] Yager DR, Kulina RA, Gilman LA (2007). Wound fluids: a window into the wound environment?. Int J Low Extrem Wounds.

[34] Trengove NJ, Stacey MC, McGechie DF, Mata S (1996). Qualitative bacteriology and leg ulcer healing. J Wound Care.

[35] Raja JM, Maturana MA, Kayali S, Khouzam A, Efeovbokhan N (2023). Diabetic foot ulcer: a comprehensive review of pathophysiology and management modalities. World J Clin Cases.

[36] Pastar I, Stojadinovic O, Yin NC (2014). Epithelialization in wound healing: a comprehensive review. Adv Wound Care (New Rochelle).

[37] Naveen N, Rajesh KN, Chethan S, Suhas NG (2024). Collagen-based mesh in the treatment of posthernioplasty mesh infection in ventral hernias: a case series. J Clin Diag Res.

[38] Kim PJ, Attinger CE, Constantine T (2020). Negative pressure wound therapy with instillation: International consensus guidelines update. Int Wound J.

[39] Morykwas MJ, Argenta LC, Shelton-Brown EI, McGuirt W (1997). Vacuum-assisted closure: a new method for wound control and treatment: animal studies and basic foundation. Ann Plast Surg.

[40] Orgill DP, Bayer LR (2013). Negative pressure wound therapy: past, present and future. Int Wound J.

[41] Akita S (2019). Wound repair and regeneration: mechanisms, signaling. Int J Mol Sci.

[42] Sorg H, Tilkorn DJ, Hager S, Hauser J, Mirastschijski U (2017). Skin wound healing: an update on the current knowledge and concepts. Eur Surg Res.

[43] Hofmann N, Rennekampff HO, Salz AK, Börgel M (2023). Preparation of human amniotic membrane for transplantation in different application areas. Front Transplant.

[44] Werner S, Grose R (2003). Regulation of wound healing by growth factors and cytokines. Physiol Rev.

[45] Massee M, Chinn K, Lei J, Lim JJ, Young CS, Koob TJ (2016). Dehydrated human amnion/chorion membrane regulates stem cell activity in vitro. J Biomed Mater Res B Appl Biomater.

[46] Eming SA, Krieg T, Davidson JM (2007). Inflammation in wound repair: molecular and cellular mechanisms. J Invest Dermatol.

[47] Gould LJ (2016). Topical collagen-based biomaterials for chronic wounds: rationale and clinical application. Adv Wound Care (New Rochelle).

[48] Brem H, Tomic-Canic M (2007). Cellular and molecular basis of wound healing in diabetes. J Clin Invest.

[49] Falanga V, Sabolinski M (1999). A bilayered living skin construct (APLIGRAF) accelerates complete closure of hard-to-heal venous ulcers. Wound Repair Regen.

[50] Kahle B, Hermanns HJ, Gallenkemper G (2011). Evidence-based treatment of chronic leg ulcers. Dtsch Arztebl Int.

[51] Moore K (1999). Cell biology of chronic wounds: the role of inflammation. J Wound Care.

